# Synthesis of Knowledge on Marine Biodiversity in European Seas: From Census to Sustainable Management

**DOI:** 10.1371/journal.pone.0058909

**Published:** 2013-03-18

**Authors:** Bhavani E. Narayanaswamy, Marta Coll, Roberto Danovaro, Keith Davidson, Henn Ojaveer, Paul E. Renaud

**Affiliations:** 1 Scottish Association for Marine Science, Scottish Marine Institute, Oban, Argyll, United Kingdom; 2 Institut de Ciències del Mar, Scientific Spanish Council (ICM-CSIC), Barcelona, Spain; 3 Department of Life and Environmental Sciences, Polytechnic University of Marche, Ancona, Italy; 4 Estonian Marine Institute, University of Tartu, Pärnu, Estonia; 5 Akvaplan-NIVA, Fram Centre for Climate and the Environment, Tromsø, Norway; 6 The University Centre in Svalbard, Longyearbyen, Norway; Aristotle University of Thessaloniki, Greece

## Abstract

The recently completed European Census of Marine Life, conducted within the framework of the global Census of Marine Life programme (2000–2010), markedly enhanced our understanding of marine biodiversity in European Seas, its importance within ecological systems, and the implications for human use. Here we undertake a synthesis of present knowledge of biodiversity in European Seas and identify remaining challenges that prevent sustainable management of marine biodiversity in one of the most exploited continents of the globe. Our analysis demonstrates that changes in faunal standing stock with depth depends on the size of the fauna, with macrofaunal abundance only declining with increasing water depth below 1000 m, whilst there was no obvious decrease in meiofauna with increasing depth. Species richness was highly variable for both deep water macro- and meio- fauna along latitudinal and longitudinal gradients. Nematode biodiversity decreased from the Atlantic into the Mediterranean whilst latitudinal related biodiversity patterns were similar for both faunal groups investigated, suggesting that the same environmental drivers were influencing the fauna. While climate change and habitat degradation are the most frequently implicated stressors affecting biodiversity throughout European Seas, quantitative understanding, both at individual and cumulative/synergistic level, of their influences are often lacking. Full identification and quantification of species, in even a single marine habitat, remains a distant goal, as we lack integrated data-sets to quantify these. While the importance of safeguarding marine biodiversity is recognised by policy makers, the lack of advanced understanding of species diversity and of a full survey of any single habitat raises huge challenges in quantifying change, and facilitating/prioritising habitat/ecosystem protection. Our study highlights a pressing requirement for more complete biodiversity surveys to be undertaken within contrasting habitats, together with investigations in biodiversity-ecosystem functioning links and identification of separate and synergistic/cumulative human-induced impacts on biodiversity.

## Introduction

“We have a catalogue of all the celestial bodies our instruments can detect in the universe, but we ignore how many living beings share the Earth with us”, so said, ecologist Robert May in 1992 (see also [1]). Biodiversity is the degree of variation that exists among “living beings” and can be defined by genetic, species or habitat factors, with consensus being that maintaining biodiversity in all its forms is fundamental to the future health of the planet [2], [3]. In fact, biodiversity is not just an important element of natural ecosystems, it is of overarching importance both scientifically and for society [4], [5], being critical to the understanding of biogeographic patterns, evolutionary history, ecosystem functioning [6], [7], and to ecosystem services and resources, which provide monetary, recreational or other values [8].

Humans have long had a great curiosity about the sea, with evidence of human study of the marine biota in European Seas existing from the 3^rd^ century B.C. [9]. Formal scientific studies started in the 18^th^ Century in the Mediterranean Sea and early 19^th^ Century elsewhere in Europe [10], [11]. These and subsequent studies have generated an abundant archive of semi-quantitative information [12]–[14], although systematic collections and descriptions of marine biodiversity have a substantially shorter history.

In the 20 years since May's statement, our understanding of marine diversity and ecosystem function has increased exponentially [15]. The Census of Marine Life (CoML), a decadal global programme from 2000–2010, greatly increased our knowledge of marine biodiversity and contributed enormously towards the investigation of the “known, unknown and unknowable” biodiversity throughout the World's oceans [16]. However, it is clear that most of the marine biodiversity still remains unknown and recent estimates vary in the time it will take to complete gain this knowledge, with estimates ranging from ∼100 years [15] to >1000 years [17].

The knowledge of the biodiversity of European Seas, which contain some of the historically and presently best explored marine areas of the world, has been substantially improved in recent years [9], [18]–[20]. Current European marine biodiversity studies include some of the most extensive investigations into the description, production and maintenance of biodiversity patterns, as well as quantification of the consequences of changes in biodiversity for system sustainability and production of ecosystem goods and services [21]–[24]. In addition, increasing efforts are being made to map and predict species occurrence and distribution using available (and most of the time, imperfect) data (e.g. [25], [26]). Current understanding, therefore, relies on synthetic efforts that bridge sampling methodologies from nets to remote sensing, perspectives from genetic analyses to habitat mapping, and analytical techniques from novel experimental design to innovative statistical models. However, there is a need to synthesise the existing knowledge in order to provide an overview of current and future challenges, identify existing gaps and provide information that is useful for a sustainable management of marine biodiversity in European Seas.

In this work we have considered the species sub-component of biodiversity and used both new and existing databases in European Seas to address the following questions: (1) what is currently known and unknown regarding marine biodiversity, (2) what is the role and importance of biodiversity in the functioning of marine ecosystems, (3) which are the anthropogenic threats to biodiversity and what implications do they have for the goods and services that biodiversity provides, and (4) how can such a synthesis of current regional biodiversity information contribute to satisfying Europe-wide management directives.

**Figure 1:**
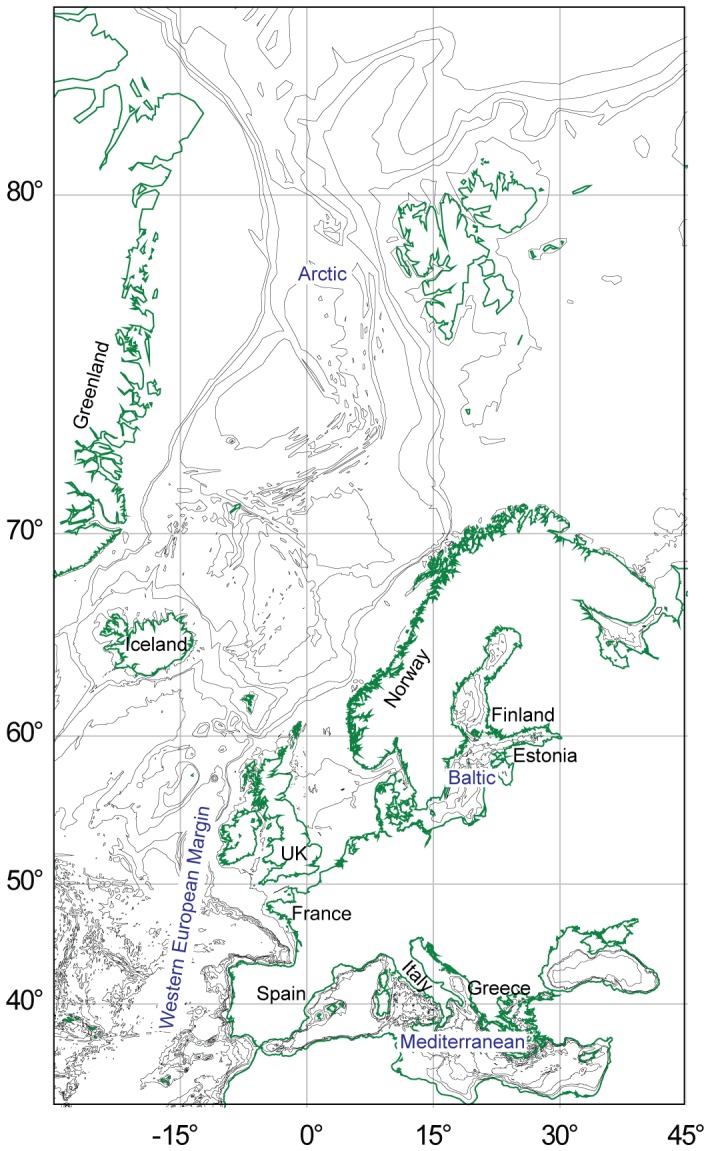
The major European Regional Seas.

We chose the European Seas (defined here as four sub regions: the European Arctic, the Western European Margin, and the Baltic and the Mediterranean Seas. Other seas, e.g. the Black Sea were not included as they were originally excluded from the CoML Mediterranean regional assessment) (Figure 1) as our area of interest as these are globally well studied regional seas [9], [18]–[20]. Moreover, in this region there is an established dialogue between biodiversity science and policy, and a growing will to inform policy using biodiversity indicators. In fact, biological diversity is the first of the 11 descriptors of Good Environmental Status (GES) on the European agenda for assessment and management of marine ecosystems, the Marine Strategy Framework Directive (MSFD) [27], and it is a fundamental component of the EU Habitats Directive (92/43/EEC) as well as for the Baltic Sea Action Plan of the Helsinki Commission (HELCOM BSAP).

## Results and Discussion

### Synthesis of the known and unknown

**Figure 2:**
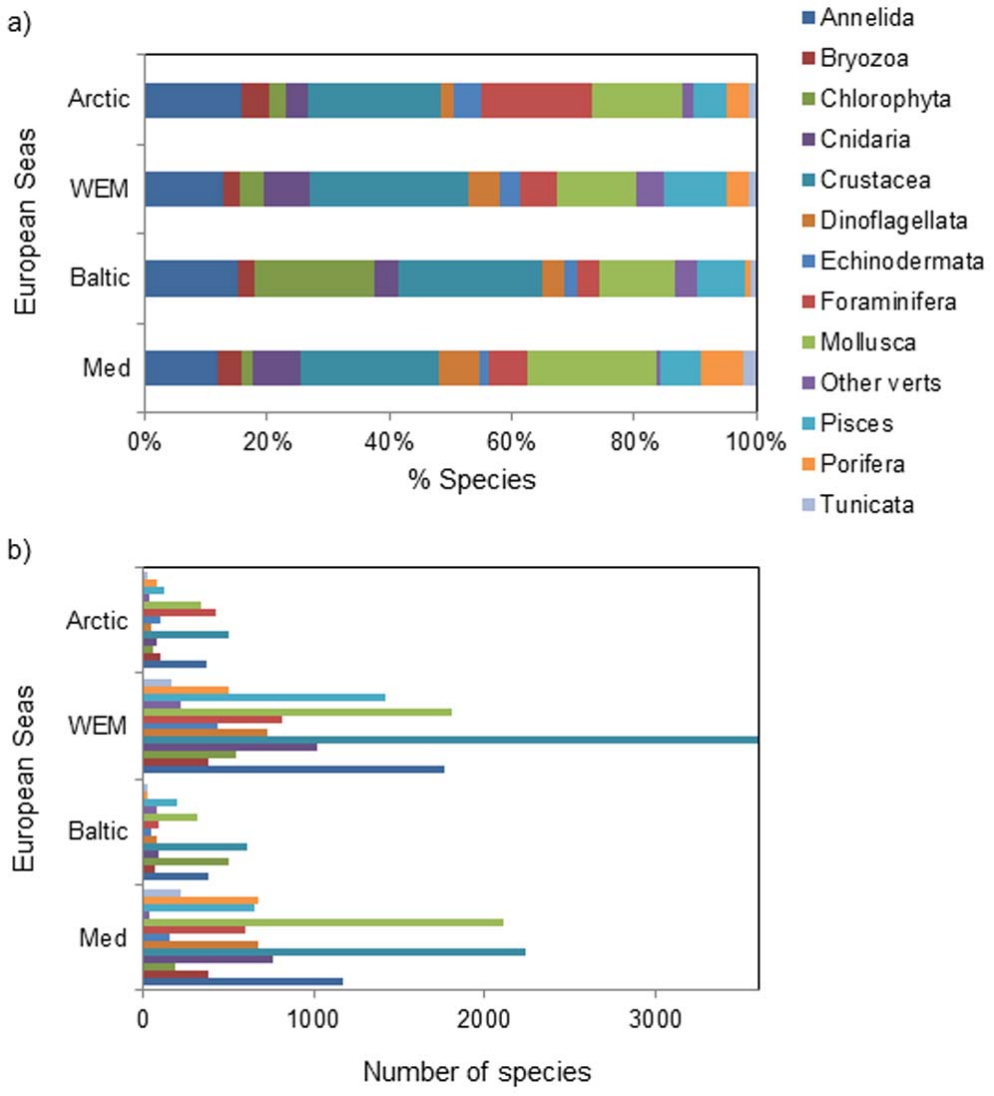
Comparison of species in different taxonomic groups in all four regions investigated. A) The percentage accumulation of species found per taxonomic group in each region (Arctic, Western European Margin, Baltic and Mediterranean Sea); B) The total number of species found per taxonomic group in each region (Arctic, Western European Margin, Baltic and Mediterranean Seas). (Sources: Coll et al. [9], Danovaro et al. [18], Narayanaswamy et al. [19], Ojaveer et al. [20]).

To date, more than 26,000 unique species are known for all European Seas excluding bacteria and viruses [28], but more than 30,000 if they were included ([29], with ∼16,000 along the Western European margin, ∼17,000 in the Mediterranean, ∼6,000 in the Baltic and ∼2,500 in the European Arctic (Figure 2a, b) [9], [18]–[20], [30]. However, the heterogeneity of the marine environment as well as the variability in life habits across the domains of life creates significant challenges in collecting and documenting marine biodiversity [31].

**Figure 3:**
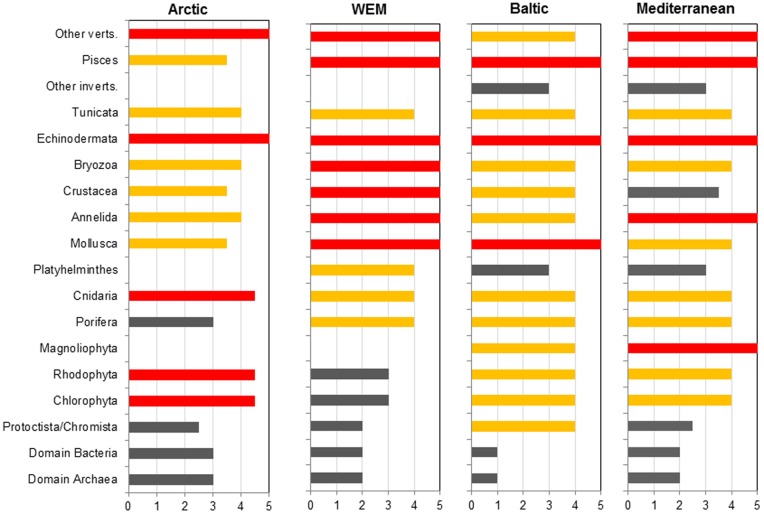
State of knowledge of taxonomic groups ranked from 1–5. Rankings equate to: 5 =  very well known; 4 =  well known; 3 =  poorly known; 2 =  very poorly known; 1 =  unknown (Sources: B Bluhm pers. comm; Coll et al. [9], Danovaro et al. [18], Narayanaswamy et al. [19], Ojaveer et al. [20]).

When assessing each taxonomic group for each region on a scale from 1 – 5 in order to determine what the state of knowledge is for each group, we observed that fish, other vertebrates and echinoderms were the groups that were most well known in all seas (Figure 3). Along the Western European Margin several of the invertebrate taxonomic groups were very well known (e.g. the crustaceans and bryozoans), whilst annelids and molluscs were well known from all the regions (Figure 3). On the contrary, the prokaryotic biodiversity and their biogeographic patterns (i.e. Bacteria and Archaea) could not be properly assessed as they are poorly known from most of the European regions.

### Microbial diversity

Microbes are ubiquitous in the sea, in both the pelagic and benthic environment. An upper limit of species abundance of 2×10^6^ globally was suggested by Curtis et al. [32], however, the absolute diversity of prokaryotes is widely held to be unknowable, and this also applies to European Seas (Figure 3). Similar uncertainty exists with respect to marine photosynthetic protists. Medlin and Kooistra [33] suggested that we have identified less than 10% of these organisms worldwide. Differences in the methodologies, types of studies and the continuously improving state of our knowledge of marine microbial diversity makes it difficult to provide full species estimates and establish comparisons. For example, last estimates highlighted that >25% of all known biodiversity species corresponded to prokaryotic (Bacteria and Archaea) and eukaryotic (Protists) marine microbes in the Mediterranean Sea, the European Arctic and along the Western European Margin, whereas in the Baltic this figure was >45% [9], [19], [20], [30]. But the data available for Bacteria, Archaea, and Protists were very limited; therefore these estimates must be treated with caution.

**Figure 4:**
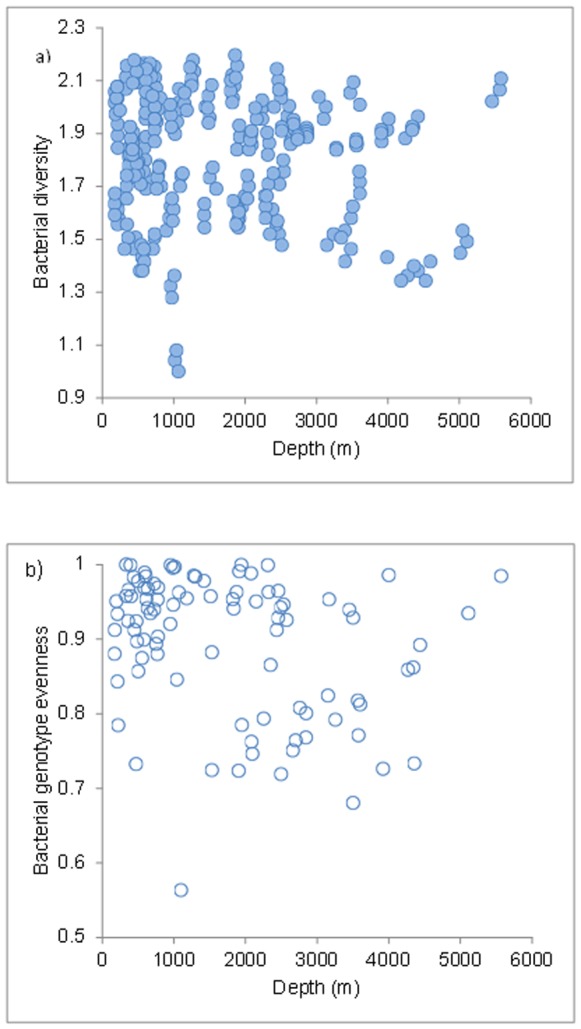
Spatial patterns of bacterial diversity as genotype richness. Reported are bathymetric gradients of A) bacterial diversity and B) Bacterial Evenness (ARISA). Data are from the Arctic (n = 42), North Atlantic (n = 33), central Atlantic (n = 100) and Mediterranean Sea (n = 113) (Sources  =  HERMIONE project and the Pangea database).

Interestingly enough, the most recent data reported in this study from the Arctic, North and Central Atlantic and Mediterranean Sea indicated that bacterial diversity (expressed as genotype richness, which is expected to provide a trend for the most represented putative taxa) does not change significantly with increasing water depth (Figure 4). However, different taxa are present in different systems and different regions are characterised by a different biodiversity [34]. Considering this high uncertainty in abundance (and distribution) it is unsurprising that the factors controlling microbial biodiversity, their major and multiple different roles in biogeochemical cycling, and as a biological resource to humans still remains largely unknown. Some preliminary results suggest that there may be a link between bacterial biodiversity and biogeochemical processes in coastal lagoons (e.g. Venice lagoon) but the relationship could be habitat specific [35]. Given their environmental diversity and natural variability, together with advanced infrastructure and human resource, European Seas are likely to be significant in advancing our understanding of how microbes support ecosystem structure. Through the development of molecular taxonomic techniques, one of the key hypotheses in microbial ecology “everything is everywhere, but the environment selects” [36] is now being challenged [37]. Another missing point relates to the interactions between microbial and faunal diversity and their interactive functional roles.

### Animal diversity

The diversity knowledge of multi-cellular animals in European Seas is higher in comparison to microbes (Figures 2a, 3 and 4). However, in the pelagic environment, the number of identified species is only well known for six groups, with <1000 species each of fish and opisthobranchs, <70 species each of euphausiids and chaetognaths, <50 species of mammals, and even the most diverse group, the Calanoid copepod, have less than 3,000 species identified [38]. In contrast to the pelagic zone, greater heterogeneity of habitats generates higher benthic biodiversity (Figure 2a).

Within a given taxon, regional-independent patterns exist across all four regions of European Seas (Figure. 1) with differing numbers of multi-cellular animal species per taxonomic group (Figure 2a, b). However, crustaceans have the greatest species richness, with almost twice the number of species compared to other taxonomic groups; followed by the annelids and molluscs. In addition, in the Baltic Sea, higher numbers of Platyhelminthes species are present, and in the European Arctic there are higher numbers of Foraminifera species. However, it is unclear whether there are actually more species in a given taxon e.g. Crustacea especially along the Western European Margin, or whether this is an artefact of sampling and/or available knowledge (Figure 3).

### Bathymetric pattern of biodiversity across different habitats

Although numerous discussions have taken place regarding patterns of biodiversity with depth, the patterns themselves are not clear. In the Mediterranean, for example, there has been some documented evidence of decreasing diversity with increasing depth for invertebrate and fish species ([9] and references therein) and, in general, biodiversity is concentrated in coastal areas and continental shelves, mainly above 200 m depth. However, these patterns did not necessarily show a monotonic decrease with depth and clear exceptions to the pattern of decreasing diversity with depth were also documented [9]. Along the Western European Margin, there are varying trends with depth dependent on the faunal group being studied as well as the location. The pattern ranges from no change in diversity through to sometimes an exponential or monotonic decrease with increasing depth ([19] and references therein).

**Figure 5:**
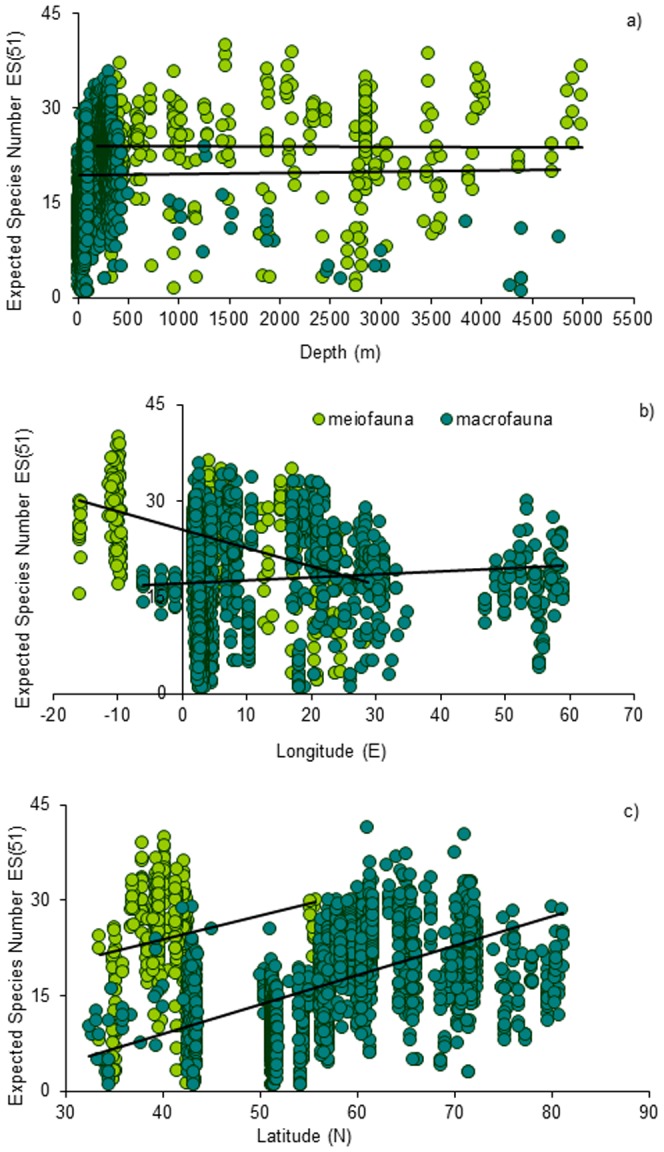
Spatial patterns of meiofaunal and macrofaunal diversity as ES(51). Reported are A) bathymetric gradient, B) longitudinal and C) latitudinal. The equations of the fitting lines are: y = −5e^−05×^+24.1 (n = 306, R^2^ = 7e^−0.5^, ns for meiofauna, y = 0.0002×+19.4 (n = 2226, R^2^ = 7e^−05^, ns for macrofauna along the bathymetric gradient; y = −0.29×+25.5 (n = 306, R^2^ = 0.18, p<0.01) for meiofauna, y = 0.05×+17.15(n = 2789, R^2^ = 0.07, ns) for macrofauna along the longitudinal gradient; y = 0.37×+8.9 (n = 306, R^2^ = 0.04, ns) for meiofauna, y = 0.46×−9.3 (n = 2821, R^2^  = 0.30, p<0.01) for macrofauna along the latitudinal gradient (sources for macrofaunal data: Rees et al. [39], Kröncke et al. [78],.new data for the deep Mediterranean Sea and Atlantic margin; meiofauna Danovaro et al. [41] and 108 additional sites from the Mediterranean and Atlantic Margins). Overall 3130 sampling sites were assembled covering the European margins from continental shelves to the deep-sea floor (down to *ca*. 5000 m depth).

When merging together the available data with that of new data generated (collected through the Italian RITMARE and EU HERMIONE projects), giving.a total of.>3000 biodiversity records for European Seas,.the results.revealed no evidence of a decline in meiofauna with increasing water depth, whilst the macrofauna displayed a general trend of decreasing diversity below 1000 m (Figure 5a). However, there was a clear change in species composition across the different bathymetric zones, indicating that biodiversity may be strongly influenced by different forcing factors and affected by multiple mechanisms/processes that act at different spatial scales. This is also evident from the analysis of biodiversity patterns at regional scales where different variables appears to be the main drivers of biodiversity distribution (see discussions in [9], [18], [19], [39]). Overall, species number decreased by almost 50% with increasing depth, however, different faunal groups decreased at different rates with increasing water depth so that the contribution of the smaller meiofauna to the overall diversity was found to increase. Below 2000 m, foraminiferal diversity increased by 20–30% whilst meiofaunal diversity increased by 60–80% [18].

Within a single region, benthic biodiversity of different faunal size groups can vary differently across different habitats. For example, significantly higher megafaunal diversity was observed in deep-sea canyons than on open slopes in the Mediterranean (Table 1), but this pattern does not hold for foraminifera and meiofauna, which displayed similar values along open slopes and canyons [18]. All benthic components investigated displayed lower values in the deep basin than along slopes and canyons. For cold-water corals, the complex structure provided by the frame-building species provides refuges for many species and increases habitat heterogeneity, creating a suitable environment for recruitment and growth of many other species [40]. This is confirmed by the large number of megafaunal species (comparable to that of slopes) and by the extremely high values of meiofaunal (as Nematoda) diversity around coral reefs.

**Table 1:** Biodiversity of different deep-water habitats in the Mediterranean Sea.

Ecosystem	ForaminiferaES (100)	Meiofauna ES(100)	Macrofauna ES(100)	Megafauna Species Richness
Slope	31.1±2.2	42.4±1.7	23.3±2.0	109±2.6
Canyon	27±4	39.7±2.2	n/a	187±0.8
Deep-water corals	n/a	48.5±1.1	n/a	129
Basin	n/a	30±2.3	8.4±1.1	n/a

The habitats were the slope, canyon, deep-water corals and deep basin ecosystem (ES100) as expected species number for 100 individuals) (source: Danovaro et al. [18]).

### Meta-analysis of latitude and longitude

Although spatial patterns of biodiversity are poorly known, regional studies have shown clear gradients of biodiversity by latitude and longitude. For example, results for the deep waters of the Mediterranean Sea showed a clear longitudinal biodiversity gradient that also occurred along the open slopes, where values decreased eastward, from Catalonia to the margins of southern Crete [9], [18]. Species distribution models also predicted clear patterns of declining biodiversity with longitude (e.g. moving from the Western to the Eastern Mediterranean Sea) and some declining trends from North to South following a latitudinal transect [9].

The new meta-analysis we conducted on pan-European deep-water datasets revealed the high variability of species richness (as Expected Species Number) in a random sample of 51 individuals [ES(51)] for both meiofauna and macrofauna at all longitudes and latitudes (Figure 5b-c). Nematodes dominated the meiofauna, their biodiversity varied longitudinally (Figure 5b), and it decreased from the Atlantic to the Eastern Mediterranean. These findings are consistent and expand those previously reported for longitudinal patterns across the Mediterranean basin and related to food quality and quantity [41]. In the Atlantic, diversity of both meiofauna and macrofauna generally increased with latitude (30° to 82° N) (Figure 5c). Interestingly, latitudinal patterns for meiofauna and macrofauna were very similar indicating that both components responded to the same environmental drivers. These patterns are likely due to the increasing productivity of the northern European regions, and a similar pattern was also reported within the Mediterranean basin, where the northern portion is richer due to the higher river and nutrient input. However, Renaud et al. [42] found little evidence of any latitudinal pattern for the shelf infauna, from the Mediterranean through to Frans Josef Land. These findings highlight the importance of analysing large data set, which enable the identification of patterns which are not always evident on analyses at smaller spatial scale.

### Importance of marine biodiversity to ecosystem functioning

**Figure 6:**
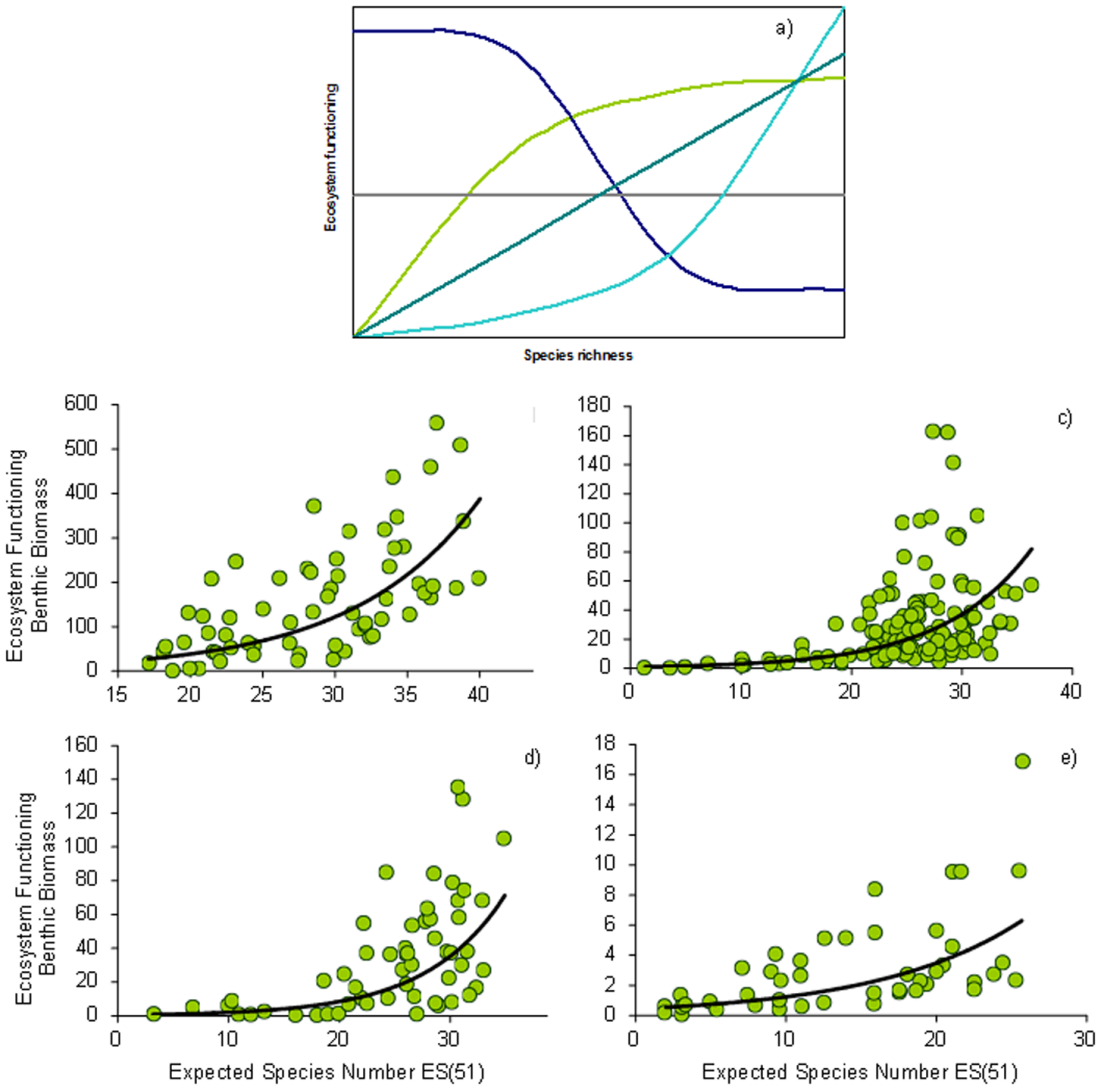
Marine biodiversity, functional diversity and ecosystem functioning. A) Conceptual diagram drawn using data from the North-Eastern Atlantic and Mediterranean Sea (Dark green: positive linear relationship between biodiversity and ecosystem functioning indicating the presence of functional complementarity among species; Light green: saturating relationship between biodiversity and ecosystem functioning indicating functional redundancy; Purple line: non-linear negative relationship between biodiversity and ecosystem functioning indicating a possible effect of selection of the few best performers; Blue line: positive and exponential relationship between biodiversity and ecosystem functioning indicating a positive interspecific interaction, such as facilitation)..([22], [43], [80]). (B-E) The relationship between expected species number [ES(51)] and ecosystem functioning (as benthic biomass µgC 10 cm^-2^) for nematodes are based on European data from Danovaro et al. [41] with new data from 198 sites from the Atlantic margins and Mediterranean Sea. The equation of the fitting lines are: b) y = 3.84e^0.12×^(n = 64, R^2^ = 0.41, p<0.01) in the North-east Atlantic; c) y = 0.44e^0.10×^(n = 45, R^2^ = 0.45, p<0.01) in the Eastern Mediterranean; d) y = 0.50e^0.14×^(n = 55, R^2^ = 0.46, p<0.01) in the Central Mediterranean and e) y = 0.86e^0.13×^(n = 128, R^2^ = 0.52, p<0.01) in the Western Mediterranean.

Biodiversity is tightly linked to ecosystem functioning, which in turn regulates the ecosystem services supplied to humans. In general, higher species richness leads to greater biomass accumulation and resource use within trophic levels [43]. In marine systems this can be observed through direct and indirect effects of biodiversity on system productivity and stability [44]. The complexity of these linkages is mediated through food-web interactions, and can produce feedback loops whereby system functioning can impact biodiversity itself [44], [45]. Based on meta-analyses of a large and varied data set, Worm et al. [8] concluded that biodiversity loss negatively impacts critical functions of marine ecosystems, including provision of food, water quality, and ability to recover from perturbations. Similarly, ecosystem resilience to natural and anthropogenic impacts has also been shown to be enhanced by higher levels of genetic, species, and functional biodiversity (e.g. [46]–[49]). A global scale study conducted in deep-sea ecosystems for the first time found empirical evidence from the real world that the rates of ecosystem processes and their efficiency are exponentially related to biodiversity, so that even a minor diversity loss can lead to a dramatic decrease of ecosystem functions and thus to the collapse of ecosystem services [22]. These findings have been confirmed by subsequent investigations conducted on tropical habitats and it can be hypothesised that similar interactions occur in biodiversity-hotspot ecosystems. Here we report new results of biodiversity data from different European Seas showing the presence of exponential relationships of species biodiversity with ecosystem functions (Figure 6). These relationships were consistent across latitudes and longitudes for all areas investigated (Eastern Atlantic margin, Western, Central and Eastern Mediterranean; Figure 6a–e). These findings, not only confirmed the importance of biodiversity in maintaining ecosystem functioning in these regions but also provide new insights into the BEF relationships in European Seas. In fact, the equations of the relationships changed notably from system to system. In particular, the Atlantic Ocean (y = 3.84e^0.12x^, R^2^ = 0.41) and the Central Mediterranean (y = 0.50e^0.1423x^, R^2^ = 0.46) displayed, for equal biodiversity levels, significantly higher values of ecosystem functioning (expressed as benthic biomass) than the Eastern Mediterranean (y = 0.44e^0.10x^, R^2^ = 0.45). If these equations can be used to predict the impact of biodiversity loss on ecosystem functions [43], then we can hypothesise that a local species extinction of the same magnitude can have a higher impact on the Central Mediterranean and Atlantic Ocean than in the Eastern Mediterranean. If confirmed, this finding could have important implications for planning strategies of biodiversity conservation and prioritizing the protection of different marine regions..Positive exponential relationships of diversity with benthic biomass highlight that even small declines of biodiversity may result in large reductions in ecosystem functioning, thus potentially prejudging the sustainable functioning of those ecosystems displaying a biodiversity loss.

This consistency between different large-scale studies gives strong support to the hypothesis that the maintenance of high levels of biodiversity is necessary to ensure ecosystem function, and in turn, security of ecosystem goods and services for humans. Elaboration of the links between biodiversity and function provide a mechanistic approach to how ecosystem services are provisioned, and importantly, which ecosystem properties should be monitored and mitigated. This, therefore, is a major issue of increasing and significant importance for environmental management of European Seas: to determine the link between biodiversity and good environmental status with ecosystem functioning and provisioning of ecosystem services to humans.

### Threats to biodiversity and their consequences for good and services

Multiple human uses such as resource exploitation, habitat destruction, pollution, nutrient loading, and alien species invasions have resulted in present day European Seas looking quite different from their original states (e.g. [14], [31], [50]). Uncertainty regarding the current state of impact, the potential changes in the ecological drivers of biodiversity, and how they might affect different faunal groups makes comparison between time periods problematic. The use of a relative threat index, however, minimizes these issues [31].

**Figure 7:**
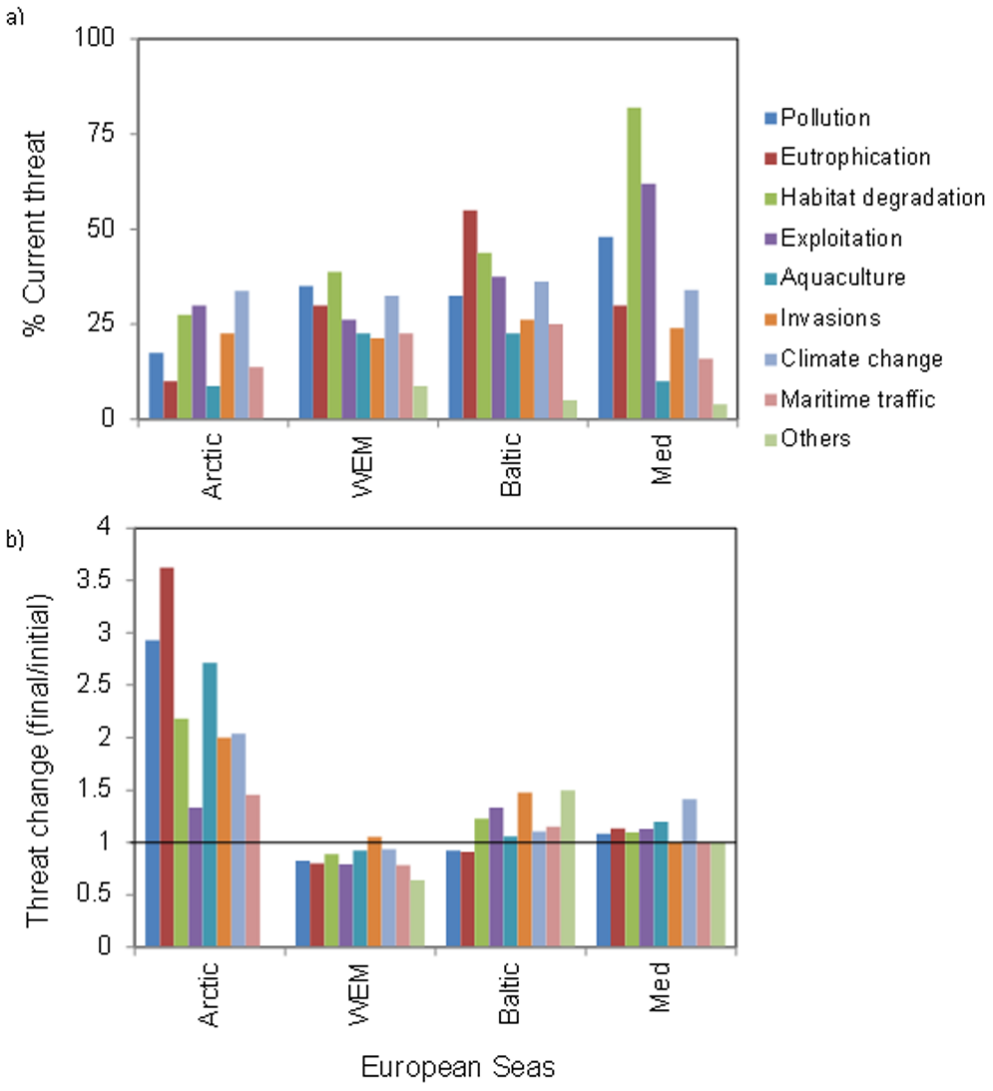
Qualitative ranking of potential threats to biodiversity from natural and human impacts. Threats were assessed for both A) current conditions and B) expected conditions in 10 years: the average value for each future threat was subtracted from the current perceived threat for comparison among regions. A negative value in the plot suggests predicted lessening of the threat (modified from Coll et al. [9] and Costello et al. [31]).

New analysis of these indicators shows that biodiversity is threatened by similar drivers across Europe's regional seas (Figure 7a). While biodiversity threats caused by increased nutrients are mainly expressed in primary producer groups, this together with overall shifts in community structure, composition and increased biological productivity, habitat degradation and species invasions effects until now have mostly been confined to benthic invertebrates. Impacts associated with climate change are observed across the organism groups from bacteria to marine birds. Primary invasions and range extensions of the already existing alien species can increase biodiversity, both species and functional diversity [51], but with unknown consequences for ecosystem function and goods and services provisioning. The current intensity of anthropogenic threats to biodiversity is geographically variable, with larger threats present in the enclosed seas (Mediterranean and Baltic Seas, Figure 7a).

Future threats, however, may evolve differently (Figure 7b). Relative to current levels, the Arctic, and to a lesser extent the Western European Margin are expected to see the greatest increase in threats over the next decade. Although an increase in threat is predicted for the already heavily impacted Mediterranean and Baltic Seas, this is somewhat more modest compared to the Arctic. However, these “modest increases” are still likely to cause further detrimental impacts to biodiversity (e.g. [25], [52]). The impact that changes in biodiversity will have on ecosystem goods and services cannot be easily predicted, but the links between biodiversity with ecosystem functioning and system resilience described above suggest that future marine ecosystems throughout Europe will present new management challenges. It is important to highlight that separate, interactive and cumulative effects of different threats on biodiversity are still poorly understood [9], [18]–[20]. For instance, climate change, together with bio-invasions, could result in a dramatic turnover of the present biodiversity, which has the potential to disrupt ecosystem services by altering the functions of the biological assemblages [53]. In the past few decades there has been a dramatic increase in the number of invasive alien species. Rilov et al. [54] suggested that not only species loss, but also changes in species composition could be potentially detrimental to the provision of ecosystem services in European Seas (particularly the Mediterranean and Baltic Seas) with respect to alien species. Species turnover, new food webs and biotic interactions may occur across Europe, but will possibly be crucial in the Arctic due to its relatively un-impacted current state, and because stressors that are now commonplace at lower latitudes such as petroleum-related pollution, fishing damage especially through trawling, and tourism are soon expected to significantly impact this location [55].

Therefore, the way anthropogenic threats will interact and accumulate in the future is a key scientific issue that needs further and urgent assessment in all European Seas. Ecological modelling applications are ideal tools to advance our understanding of how cumulative and interactive effects of multiple drivers may affect marine biodiversity and ecosystem functioning. Various applications have been developed in European Seas (e.g. [56]), and exponential development is foreseen for the near future [57]. Parameterization of these models, however, relies on continued advances in biodiversity knowledge and understanding the ecological mechanisms linking biodiversity and ecosystem functioning. This synthesis suggests that a regional sea 'case-study' approach is a valuable first step within European Seas, after which integration of results into an informed management framework can proceed.

### Marine biodiversity and European environmental management

#### Current initiatives

The key piece of European legislation on the marine environment, the Marine Strategy Framework Directive (MSFD), identifies biological diversity as an important descriptor to ensure Good Environmental Status (GES) in European Seas. Specifically, biodiversity assessment is required at several ecological levels, ecosystems, habitats and species [58] to confirm that “*Biological diversity is maintained. The quality and occurrence of habitats and the distribution and abundance of species are in line with prevailing physiographic, geographic and climatic conditions”.* Several other GES descriptors and related assessment requirements, such as ecosystem functioning include marine biodiversity implicitly or explicitly [27], [58].

However, the evaluation of biodiversity status and its threats, however, remains problematic. For example, the MSFD eutrophication task group [59] noted that research was still needed on how to evaluate the optimal extent and status of marine habitats to support viable and diverse communities and the valuation of goods and services they provide. This creates substantial challenges not only for EU member countries but also regional sea conventions, such as the Oslo-Paris Convention (OSPAR) (NE Atlantic), the Helsinki Commission (HELCOM) (Baltic Sea) and the Barcelona Convention (Mediterranean Sea). In addition, there is also a role for international scientific organisations like the International Council for the Exploration of the Sea (ICES) or the International Commission for the exploration of the Mediterranean Sea (CIESM) to further advance knowledge pool of marine biodiversity science and advice, for instance, to develop, test and suggest new indicators, and investigate links between state and pressure indicators (e.g. [60]).

Scientific and management communities have devoted considerable effort over the past decade to identify threatened and declining species and habitats, characterize the extent of the threat/decline, and evaluate associated ecosystem consequences (e.g. [25], [61]). Most of the studies dealing with threatened/declining species relate to upper trophic levels, and the findings are not encouraging. For example, threat indicators based on the estimated population status of North Sea fishes using the International Union for the Conservation of Nature (IUCN) Red List decline criteria, suggested that the proportion of threatened fishes and the degree of threat had increased steadily over time, with all species since the late 1990s meeting the “vulnerable” criterion [62]. In the Baltic Sea, all four marine mammals and 34 fish (∼18%) species have been identified as high priority for conservation ([63] and references therein). Most threatened (fish) species have low intrinsic population growth rates and suffer high fishing mortality. Species with a lower risk of extinction have either resilient life histories or are species subject to intense fisheries management [64]. Many cartilaginous fish in the Mediterranean Sea are vulnerable and several stocks have collapsed during the last century [9], [65], [66]. Thus, with regards to management, biodiversity is generally considered in terms of a relatively few (exploited) species and/or habitats (see below), while wider genetic/taxonomic/community aspects remain as yet an unachievable challenge.

An important mechanism for safeguarding the critical functions of both pelagic and benthic biodiversity is the use of Marine Protected Areas (MPAs) and these have now been established in all European regional seas. One of the main criteria for selection of MPAs is the presence of a species/habitat in need of protection (identified as threatened and/or declining species and habitats). Currently, the three major European regional marine environment management areas (i.e., Mediterranean Sea, Northeast Atlantic and the Baltic) contain >800 planned or established Special Protected Areas (SPAs) and 133 sites of Scientific Community Importance with an overall surface area of ∼124,000 km^2^ and ∼179,000 km^2^, respectively, but distribution is patchy and the proportion of spatial coverage in each area is still limited [23], [25], [67], [68]. Regional studies of biodiversity, ecosystem function, and threats, therefore, can provide one of the bases for identifying target areas for future MPAs.

There are two current major initiatives to address conservation of marine biodiversity and sustainable use in areas beyond national jurisdiction in European Seas. The Food and Agriculture Organization of the United Nations (FAO) has initiated a process to identify Vulnerable Marine Ecosystems (VMEs), and adopted international guidelines to define management frameworks to prevent significant adverse impacts on them. But, until now, measures to protect VMEs have been almost exclusively closure of areas considered to have significant concentrations of corals. Another initiative was taken by the Convention of Biological Diversity (CBD), which most recently started a regional-scale initiative to identify ecologically or biologically significant marine areas (EBSAs) through cooperation with national governments as well as relevant management or advisory organizations. The process of EBSA identification is a scientific and technical step and needs to be kept separate from the processes used to decide on the policy and management responses that are appropriate for providing the desired level of protection to those areas [69]. In addition to these two global initiatives working in European Seas, OSPAR has made considerable progress in establishing MPAs in the North Atlantic, and the Barcelona Convention has been designating SPAs in the Mediterranean areas beyond national jurisdiction.

#### New knowledge and tools for management: a regional approach

Developing a toolbox for identifying GES across European waters, as called for in the MSFD, is a daunting task and is probably best begun through a regional approach. Biodiversity itself is still unevenly described among different taxa, and regions, and knowledge syntheses can be useful in highlighting this issue (Figure 3). For example, our knowledge and understanding of the smaller sized meiofauna, which exhibit the highest number of expected species and the greatest proportion of unknown diversity, is relatively low compared to their larger macro- and mega- faunal counterparts. It is estimated that potentially >60% of the Mediterranean deep sea meiofauna still remain to be discovered [18]. The microbial diversity is completely neglected although the key role of microbial species in biogeochemical cycles is fully recognized, in symbiotic interactions and in sustaining all other life forms. Moreover, compared to species and populations, our knowledge-base on biotopes/habitats is much weaker. For instance, a critical analysis of the most recent marine habitat classification list produced for the Mediterranean Sea showed that ∼40% of habitats (and associated species) considered were scarcely covered by scientific knowledge, and generally scant quantitative information on the geographical distribution of selected habitats and associated species was available [70]. In addition, all assessed biotopes/habitats in the Baltic Sea are considered to be threatened, the conservation status of most is inadequate, and all are in urgent need of protective measures [63]. Therefore, improved inventories of marine habitats are needed to support mapping activities and spatial ecosystem-based management [71], [72].

How successful has the scientific community been in meeting the multiple challenges regarding diversity in the oceans? European Seas are some of the most well studied [14], [31], and it can be argued that Europe has been possibly the most active in establishing regional management policies. However, much still remains to be done before the ultimate target (at least 10% of all marine ecological regions to be effectively conserved [73]) will be achieved across Europe. Moreover, future conservation strategies will need revision as current MPAs potentially miss hotspots of diversity traits and anthropogenic impacts (e.g. [23], [25]).

#### Requirements to meet future challenges

Here we have attempted to synthesise the available knowledge on European Marine Biodiversity using both new data and existing databases and demonstrated significant progress in species identification and understanding of the link between anthropogenic drivers, biodiversity and ecosystem function. However, many species remain un-discovered and/or un-enumerated, with no habitat having been fully explored and hence our quantitative understanding of these links remains poor.

It is therefore clear that much still remains to be done, with major challenges to be faced in deciding what we can achieve and what information will remain unknowable. Meeting these challenges will require:

- Investing resources in taxonomy (classical coupled with molecular taxonomic studies) with specific training of new specialists also able to undertake new molecular techniques to describe cryptic biodiversity and resolve uncertainties related to the use of classical taxonomy,
- investigating species interactions, life traits, cycles and histories and populations' connectivity, of most marine species and populations across different habitats and ecosystems are still completely unknown,
- standardising methodologies in order to ensure that subsequent data and results can be rigorously and statistically compared and validated,
- increasing sampling effort and exploration of novel habitats and in identifying and mapping biodiversity hotspots,
- making both historical and new data increasingly more accessible once published,
- expanding studies of microbial diversity and on the understanding of its interactions with other biological components of the marine ecosystem,
- quantifying ecosystem services and the impact of loss of biodiversity on these goods and services in different marine habitats/ecosystems/regions, and how cumulative and synergistic anthropogenic impacts may impact these services,
- identifying and quantifying the link between the pressure on biodiversity and state indicators,
- enhancing the importance of biodiversity in marine management policy decisions.

Some of the challenges outlined above have been recently identified as a priority activity for ICES [60]. In addition to describing biodiversity at its many levels, new technology, monitoring, and analytical tools are needed to define and maintain GES in a meaningful and practical manner. Indicators of biodiversity must be developed and compared among regions, with biodiversity syntheses providing the crucial ground-truthing data. Habitat, food-web, and statistical models can be applied on a regional basis in both a descriptive manner, and in a dynamic mode whereby effects of altered biodiversity can be experimentally evaluated to answer questions regarding, e.g. effects of introduced species, impacts on ecosystem functioning, and consequences for goods and services. Preservation and maintenance of biodiversity is a complex issue requiring consideration of geographic, political, economic and social factors [74], often differing considerably across a region as diverse as Europe. Resolution of these often-conflicting pressures, however, must be reached if we are to achieve the common aim of ecosystem-based management of the marine environment [75]. Through integration of efforts to develop indicators, monitoring, and impact-assessment strategies, and identification regional and spatial threats and other human pressures, we can move closer to resolving conflicts while addressing Europe's goals of managing biodiversity in its marine waters.

## Materials and Methods

No specific permits were required for the described field studies. The field studies did not involve endangered or protected species.

### Methods for Figure 2

Here we used datasets collected and synthesised recently (sources: [9], [18]–[20]) as well as accessing new and updated results from EurOBIS ([30]) and integrated them to provide new results of biodiversity patterns at a European Sea scale.

### Methods for Figure 3

A number of taxonomic experts from each region were asked to assess each taxonomic group and rank them from 1–5, where 5 is the most well-known. This enabled us to determine the current state of knowledge for each major taxonomic group. The definitions for the rankings are as follows: 5 =  very well known (>80% described, identification guides <20 years old, and good taxonomic expertise); 4 =  well known (>70% described, identification guides <50 years old, good taxonomic expertise); 3 =  poorly known (<50% species described, identification guides old or incomplete, moderate taxonomic expertise); 2 =  very poorly known (only few species recorded, no identification guides, little taxonomic expertise); 1 =  unknown (no species recorded, no identification guides, no expertise) (sources: B Bluhm pers comm., [9], [18]–[20]).

### Methods for Figure 4

Bacterial diversity patterns were investigated using ARISA a highly reproducible fingerprinting technique commonly utilized for describing bacterial diversity distribution and the main components of the bacterial assemblages in marine environments [76], [77]. ARISA involves PCR amplification of the highly variable intergenic spacer region (ITS1) between the 16 S and 23 S rRNA genes, followed by separation and detection of the different-length products. ARISA allows to discriminate the ‘‘Operational Taxonomic Units’’ (OTUs) that differ by ca. 98% or less in 16 S rRNA sequence similarity. ARISA was preferred to 454 tag sequencing because it allowed an assessment of diversity patterns at an unparalleled sample size and spatial scales [76].

### Methods for Figure 5

Sampling was carried out in the North and Central Atlantic Ocean (100 sites), the Western and Eastern Mediterranean Sea (98 sites). These areas included continental shelves and margins as well as open-ocean sites. At all of the sites, macro- and meio- (namely nematodes) faunal samples were collected, identified to the lowest taxonomic level and species richness analyzed. Part of the biodiversity data set of from the Atlantic Ocean and Mediterranean were obtained from the literature [22], [41]. In particular, sources for macrofaunal data are Rees et.al. [39], Kröncke et al. [78].and new data for the deep Mediterranean Sea and Atlantic Margin are from the EU funded HERMIONE project; meiofauna Danovaro et al. [41]; these data were implemented in the present study with 108 additional sites from the Mediterranean and Atlantic margins. Since most species diversity indices are sample-size dependent, we applied the rarefaction method was so that all samples could be reduced to the same size, with ES(51) as the expected number of species in a hypothetical random sample of 51 individuals. All indexes of biodiversity were calculated with the PRIMER 6 statistical package [79].

### Methods for Figure 6

Ecosystem functioning [22], [43] was determined as total faunal biomass, a proxy for production. For the determination of faunal biomass, we calculated the individual biomass of all animals belonging to different taxa [80]. Nematode biomass was calculated from bio-volume (n = 100 per replicate) with Andrassy's formula (V = L × W^2^ × 0.063 × 10^−5^, in which body length, L, and width, W, are expressed in µm). For all of the other taxa, the biovolume was measured for all of the specimens encountered and was derived from measurements of body length (L, in mm) and width (W, in mm), using the formula V = L × W^2^ × C, where C is the approximate conversion factor for each meiofaunal taxon. Each body volume was multiplied by an average density (1.13 g cm^−3^) to obtain the biomass ( µg DW: µg WW = 0.25) and the carbon content was considered to be 40% of the dry weight.

### Methods for Figure 7

Here we standardized and modified the methodology used by Coll et al. [9] and Costello et al. [31] to determine the current and future threats to biodiversity from both natural and anthropogenic impacts. Following interviews with experts from the different geographical regions, a scale of 1 (low importance) – 5 (very important) was assigned to 17 different taxonomic groups for each potential threat in each region (sources: [9], [18]–[20]).
